# Pets, Genuine Tools of Environmental Pollutant Detection

**DOI:** 10.3390/ani13182923

**Published:** 2023-09-14

**Authors:** Cristina Hegedus, Luisa Andronie, Paul Uiuiu, Eugen Jurco, Eva Andrea Lazar, Silvana Popescu

**Affiliations:** 1 Department of Fundamental Sciences, Faculty of Animal Sciences and Biotechnologies, University of Agricultural Sciences and Veterinary Medicine Cluj-Napoca, 400372 Cluj-Napoca, Romania; paul.uiuiu@usamvcluj.ro; 2 Department of Biophysics, Meteorology and Climatology, Faculty of Forestry and Cadastre, University of Agricultural Sciences and Veterinary Medicine Cluj-Napoca, 400372 Cluj-Napoca, Romania; 3 Department of Technological Sciences, Faculty of Animal Sciences and Biotechnologies, University of Agricultural Sciences and Veterinary Medicine Cluj-Napoca, 400372 Cluj-Napoca, Romania; eugen.jurco@usamvcluj.ro; 4 Association for the Welfare of Horses, 725700 Vatra Dornei, Romania; lazarevaandrea@gmail.com; 5 Department of Animal Hygiene and Welfare, Faculty of Veterinary Medicine, University of Agricultural Sciences and Veterinary Medicine Cluj-Napoca, 400372 Cluj-Napoca, Romania; silvana.popescu@usamvcluj.ro

**Keywords:** sentinels, dogs, cats, environmental pollution, indoor pollution, chemical contaminants, environmental diseases, passive smoking, biologic contaminants

## Abstract

**Simple Summary:**

“Guardians of health”, our canine and feline companions, emit early warning signals of dangerous lead, cadmium, and arsenic environmental pollution. Many of the epigenetic studies in recent years have investigated the role of these pollutants in the pathogenesis of human diseases by using animal models and analyzing diseases with similar symptoms in pets and people. Not only several cancers (of the bladder, mammary glands, testes, ovaries, lymphomas, mesotheliomas, carcinomas) but also hyperthyroidism, pulmonary structural changes, fertility problems, cardiac and metabolic diseases (like obesity) are at least partly triggered by environmental factors to which we are exposed. Currently, canine and feline hair and other established biomarkers can give information on the pollution or contamination degree, especially regarding heavy metal and passive smoking exposure.

**Abstract:**

In a shared environment, our companion animals became unintended sentinels for pollutant exposure consequences, developing even earlier similar conditions to humans. This review focused on the human–pet cohabitation in an environment we all share. Alongside other species, canine and feline companions are veritable models in human medical research. The latency period for showing chronic exposure effects to pollutants is just a few years in them, compared to considerably more, decades in humans. Comparing the serum values of people and their companion animals can, for example, indicate the degree of poisonous lead load we are exposed to and of other substances as well. We can find 2.4 times higher perfluorochemicals from stain- and grease-proof coatings in canine companions, 23 times higher values of flame retardants in cats, and 5 times more mercury compared to the average levels tested in humans. All these represent early warning signals. Taking these into account, together with the animal welfare orientation of today’s society, finding non-invasive methods to detect the degree of environmental pollution in our animals becomes paramount, alongside the need to raise awareness of the risks carried by certain chemicals we knowingly use.

## 1. Introduction

Evidence that living beings can be natural sentinels of biological risks and environmental pollutants has been recognized for centuries [1]. In their book, Natterson-Horowitz and Bowers [2] point out that “In a world where no creature is truly isolated and disease spreads as fast as airplanes can fly, we are all canaries, and the entire planet it’s our coal mine”. Many birds, fish, wild and domesticated terrestrial mammals, and pets are valuable indicators of environmental pollution, displaying early warnings of exposure to a contaminated environment before humans are affected [3].

Sentinels are organisms whose characteristics (including health status) change due to acute or chronic effects in a given environment that can be evaluated (monitored) through serial surveillance [4,5,6,7]. Samples can be collected routinely, at random, or at predetermined intervals, and analyzed to identify potential health hazards to humans and other animals [4]. There are many criteria according to which a species should be considered a sentinel. In the first place, the sentinels must occupy a large area geographically close to human settlements and humans; their biology, sensitivity to pollutants, and bioaccumulation capacity have to be known [5]. They must have at least the same sensitivity to poisoning as humans, with similar physiology; their life span must be long enough to show the effects of not only the acute but of chronic exposures too, and the biological or clinical effects must develop early and be comparable to those in humans. The exposure pathway has to be similar to that of humans, which is feasible for companion animals sharing the same environment with their owners [4]. Among the phenotypic characteristics, the animals’ size is essential because of the sufficient amounts of tissue are needed for analysis, but other aspects, such as age or gender, must also be considered [6].

By testing and monitoring pets, we can detect early the presence and impact of pollutants for this information to be used to minimize the adverse effects on human health [7]. Thus, companion animals can literally be considered “sentinels” of environmental pollution [8].

The definition of sentinel organisms many times overlap with that of biomonitors, although the latter term may be considered broader. A biomonitor is an organism (or part of an organism or a community of organisms) that contains information on the quantitative aspects of the quality of the environment [9]. In this sense, sentinels are biomonitors too. Yet, active biomonitoring has a more intentional sense when, for example, biomonitors bred in laboratories are placed in a standardized manner in a certain environment to gather information. By contrast, especially when the term ‘natural sentinels’ is used, the organisms already present in the environment are monitored.

The aim of this paper was the following: (1) update information on pollutants and contaminants that affect the health status of humans and pets sharing the same environment or microenvironment; (2) present accepted non-invasive, early detection methods to identify pollutants and contaminants harmful for pets, proper for parallel human–pet epidemiological studies; (3) highlight the sentinel role of pets in the service of human medical research; and (4) raise awareness about the need to protect companion animals and ourselves from harmful environmental influences by more responsible everyday usage of certain products.

The types of pollutants chosen for this review included the following: heavy metals, asbestos, second-hand smoke, forever chemicals (persistent organic pollutants and brominated flame retardants), pesticides, insecticides, and biologic contaminants (microorganisms).

## 2. Methods

**Figure 1:**
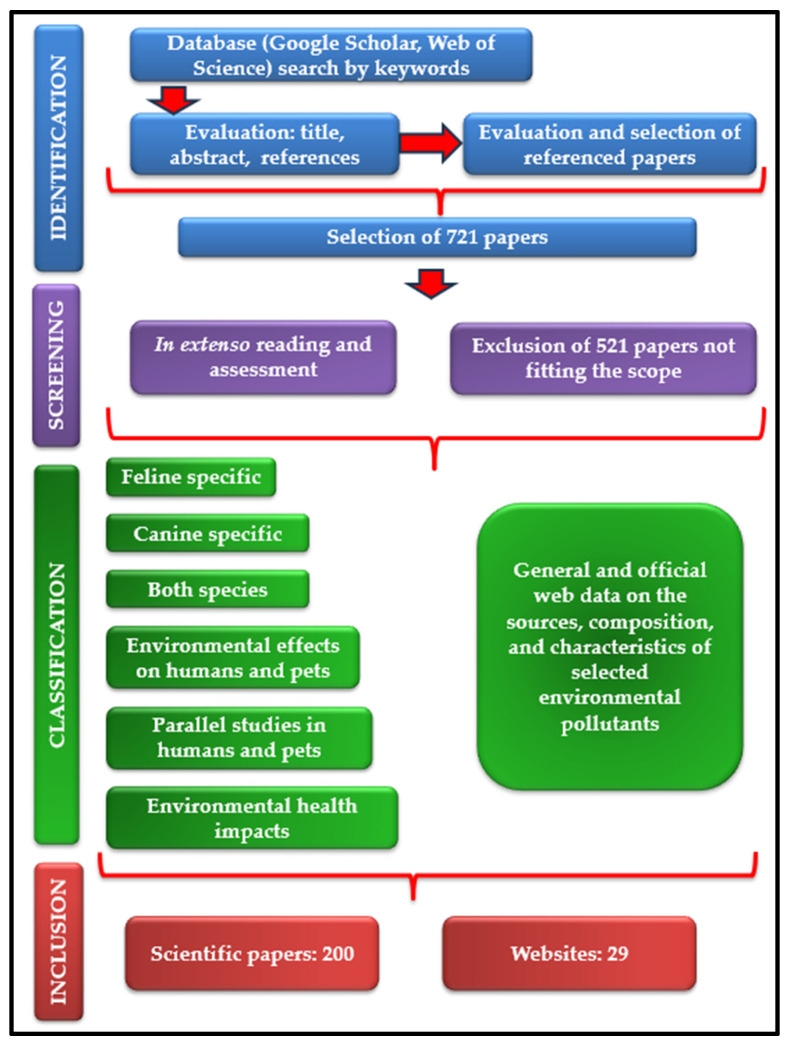
Flowchart of the review process.

In order to prepare this scientific review, a multistage methodology was performed (Figure 1). The first stage consisted in a thorough analysis of open-access scientific papers primarily sourced from Google Scholar and Web of Science platforms. Plenty of keywords have been used, such as “domestic sentinel species”, “companion animal sentinels”, “dog”, “cat”, “environmental diseases”, “environmental pollution”, “indoor pollution”, “lead pollution”, “cadmium pollution”, “arsenic pollution”, “mercury pollution”, “per-fluoroalkyl substances”, “poly- fluoroalkyl substances”, “polybrominated diphenyl ethers”, “brominated flame retardants”, “endocrine-disrupting substances”, “passive smoking”, “second-hand smoke″, “human environmental diseases”, “environmental cancer risk factors”, “environmental causes of hyperthyroidism”, “silicone tags”, “silicone collar”, “one health”, and “microbial pollution”. This stage continued with the evaluation of each title and abstract of the articles found. In addition, the cited references of the publications were examined and several articles have been selected, paying attention to their alignment with the aim of the present review. The second stage examined the manuscripts “in extenso”, for the final decision regarding their inclusion in the review. Out of the total of 721 papers selected during the first work stage, we excluded those concerning wild animals, farm animals, plants (moss, lichens), and also those studying other risk factors of the environmental diseases discussed in the review. In the end, 200 research papers were selected for inclusion. Out of these, 26 are feline specific, 79 canine specific, 28 discuss both species, 46 are focused on human health modifications after simultaneous exposure of people and pets to the same environmental pollutants or describe common human and animal health issues. A low number (8) of papers describe diseases with similar epidemiology in humans and animals revealed by parallel studies, with high relevance in the observation of early symptoms of environmental pollutant exposure in pets to prevent human pathologies. Alongside these, 13 research articles were chosen for their relevance in explaining the environment’s health impact, and 29 websites were used and referenced to complete the datasets obtained.

## 3. Pets as Sentinels for Environmental Pollution

### 3.1. Pets as Sentinels for Asbestos Fibers and Heavy Metals in the Environment

The usefulness of animals in the role of sentinels has been recognized more than a quarter-century ago. As the National Research Council of the United States devised [10] in 1991, the biologic effects of suspected toxic substances can be evaluated in animals kept in their natural habitat (including human homes) to assess the intensity of exposures, measure the effects of chemical mixtures, and determine the results of low-level exposures over a long period. Even more, observing the prevalence and incidence of certain pet pathologies reveal patterns that show the distribution of pollution in the area [11]. 

**Figure 2:**
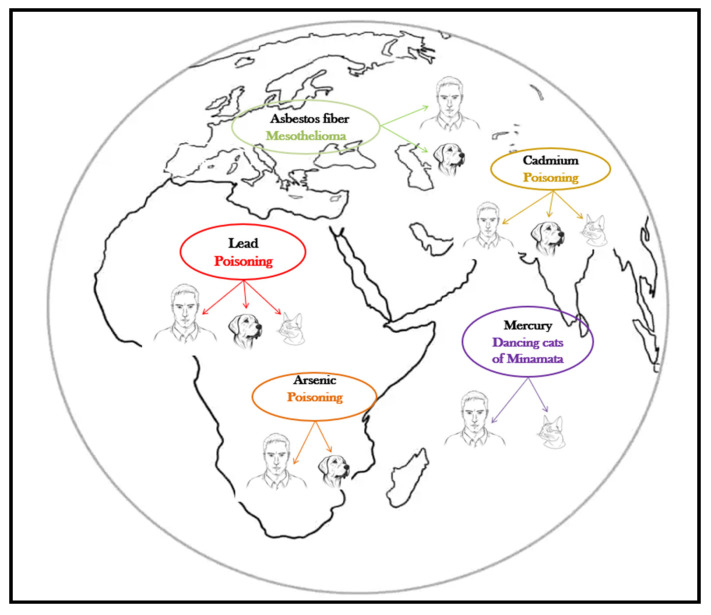
Pets, sentinels of environmental heavy metal/metalloid pollution.

The harmful effects of metalloids and heavy metals are well described, and their association with specific diseases is as well, both in humans and companion animals (Table 1, Figure 2). The harmful health impact of these pollutants is similar, regardless the species exposed [12].

Canine mesothelioma is described as being linked to lifestyle, diet, and asbestos exposure, but the most cases have occurred in canine companions whose owners worked in environments with asbestos or used flea repellents in which the talc was contaminated with asbestos [38]. The human and canine malignant mesotheliomas are clinically and morphologically similar [39], but the latency period is much shorter in dogs (8 years) than in humans (up to 20 years) [38]. As for the effects of acute asbestos exposure, in a 15-year surveillance study in search-and-rescue dogs Otto et al. [40] reported no difference in the cause of death of dogs exposed to several classes of toxicants (including heavy metals and asbestos, during deployment at terrorist attack sites) compared to unexposed dogs.

Lead (Pb), cadmium (Cd), arsenic (As), and mercury (Hg) are all heavy metals/metalloids with adverse health effects, and their latency period is also shorter in pets compared to humans [41]. The absorption, metabolization, transformations, and toxicity of these substances in pets is the most similar to that of young children, which makes companion animals even more valuable in early detection [42]. In a 2005 study, Park et al. [43] concludes that dogs can be considered biomonitors of the environmental quality assessment for cadmium, lead, and chromium contamination, and at least for lead and cadmium cats showed the same conclusive results (Table 2).

Although the concentration of heavy metals can be measured in various samples, the liver and kidney tissue are preferred, especially for cadmium and lead exposure [45,46]. Once absorbed, lead is redistributed in the soft tissues [48], but most of it (90%) is accumulated in bones and teeth [48], with a half-life of 25 years in humans [53].

The benefits of using animal sentinels to evaluate environmental lead pollution have been recognized for more than 40 years [54]. This is extremely relevant, especially when pets live in proximity to children. Preventing exposure to lead during nervous system development is very important because of the damage this heavy metal can cause [55]. The tremendous benefit of this approach is that the blood tests of sentinels can provide early warnings without having to analyze a child’s brain [4]. As early as 1976, the comparative analysis of serum samples collected from 119 children and 94 dogs from suburban Illinois demonstrated that canine companions can be considered sentinels of lead poisoning in children [54]. The presence of lead in the blood of pets may indicate potential exposure of humans, especially of young children, to the polluted environment is also the conclusion reached by Berny et al. [56]. For possible lead contamination in children, the prediction is based on canine and feline serum values which exceed the physiologic threshold of the species 2–4 times [2,57]. However, for the results to be conclusive, variables such as sex, age, and behaviors influencing exposure of the companion animals must be taken into consideration. Although the blood levels of lead are usually higher in males, Toyomaki et al. [44] reports for one of their research areas a significantly higher lead contamination of female dogs, especially in the older ones. The authors’ supposition is that male dogs may wander more (thus leaving the polluted areas), while the females stay for longer while raising their puppies in the same (polluted) place.

After the period in which the sentinel role of companion animals for environmental asbestos and heavy metal pollution detection was proven by testing their blood and especially tissues (to verify chronic exposure) illustrated by many reports (Table 2), the research continued to find more non-invasive approaches [58]. In this context, Petrov et al. [59], and Nikolovski and Atanaskova [60] believe that using hair as a testing matrix brings significant advantages in terms of ease of collection and a more animal welfare friendly attitude compared to tissue sampling. As Table 3 shows, the concentration of several heavy metals has been tested in different countries by analyzing hair samples of the companion animals exposed to these pollutants.

While testing canine hair, Jafari [61] observed a strong positive linear correlation between the contamination of the soil and the hair with chromium, copper, lead, and mercury, and to a lesser extent with zinc, and a negative correlation with arsenic. Promising results were obtained by testing feline hair too, for environmental lead pollution [69]. Aeluro and Kavanagh [69] found higher lead concentrations in the hair of female cats than of the males, but the authors report that these animals were more exposed (spent more time outdoors) compared to the males.

Similarly, to organ tissues, hair samples are the most proper when long-term exposure is suspected [70]. Through their follicles, the hairs are connected to the blood flow, extracting and incorporating not only the nutrients necessary for growth but other elements as well. Because keratin has a good metal-binding affinity, these elements become ‘trapped’ in the cortex and cuticle of the hairs [71], allowing for their testing. As opposed to blood, which is primarily a transport medium and deposits the absorbed substances readily (leading to content fluctuations), hair is a more stable medium for the identification of deposit substances such as minerals and metals [72]. By these characteristics, the hair samples reach a higher similarity relevance as the internal organs for testing chronic exposure to heavy metals irrespective of the exposure source and access ways inside the organism (whether these are air, food, or waterborne pollutants, or if they cross the skin barrier directly from the outside environment).

### 3.2. Pets as Sentinels for Heavy Metals in Water and Food

Food and waterborne heavy metal intoxications have been documented several times in the scientific literature. The pollution of surface waters with heavy metals is a concerning situation that needs special corrective measures or simply limits on the drinkability of that water source. One such situation exists in Argentina, where many regions are known as chronic regional endemic hydro arsenic zones due to the arsenic contamination of the water. A special contribution to the early detection of chronic waterborne arsenic exposure is canine hair, according to Vázquez et al. [62], with higher values (thus easier to test) than in human hair or the water itself (evidently).

Most of the pollutants in water and food come from the environment, but they significantly increase the health hazards to human and animal populations. The inhabitants of a polluted area (people and pets) will not only be exposed to the direct effect of the pollutant in their environment (from air, soil, contact surfaces) but will receive additional pollutant doses while they eat or drink. In some cases, plant- and animal-origin food is even more dangerous because of the accumulation of pollutants in these. For terrestrial or marine environmental contaminants, such as mercury accumulated in dietary products, sled dogs (*Canis lupus familiaris*) in the Arctic regions are sentinels for the local communities regarding the possible risks associated with the food they consume. The better they fit this role as mercury absorption, accumulation, and excretion in dogs are similar to humans [73,74,75]. Even the blood-to-hair mercury ratio in dogs (200) is similar to that described in humans [64]. Because there is a highly significant positive correlation between the total mercury in canine blood and hair, dogs can be reliable sentinels of mercury exposure regardless of the sampling method used for quantifying [64,68]. 

Cats suffer pathological changes from oral methylmercury poisoning similar to those produced in humans suffering from the disease called Minamata disease, discovered in 1959, but whose initial results were never published [76]. The experiment, later repeated by Eto et al. [77], involved sprinkling the cats’ food with liquid mercury, which led to observations related to the accumulation of the metalloid in the cerebrum, cerebellum, liver, and kidney. In both humans and cats, mercury poisoning has the same symptoms, the incoordination known as “dancing cats of Minamata” being an early warning sign of intoxication [78].

Most of the time, oral poisoning with mercury occurs as a result of mercury-polluted pet food. In feline companions, mercury accumulation is influenced by age and sex. In geriatric males, the accumulation begins earlier compared to females and has slightly higher values [67]. Similar to dogs, it was proven that there is a strong correlation between the mercury content in the cats’ hair and that found in their liver and kidneys after the ingestion of mercury-polluted foods. Hence, the conclusion drawn by Skibniewska and Skibniewski [65] is that feline hair can provide an indication of mercury exposure, and its concentration is correlated with the existing loads in other vital organs (liver: 0.030 ± 0.031, kidney: 0.026 ± 0.025 mg⋅kg^−1^). Wild cat hair lends itself equally well to environmental biomonitoring, according to Behrooz et al. [66].

The first alarm signal about the potential danger that threatens the integrity of human health is the accumulation of mercury values five times higher in the body of pets compared to that in the human body [79].

## 4. Pets as Sentinels for Microclimate Pollution

### 4.1. Pets as Sentinels for the Effects of Passive Smoking

**Figure 3:**
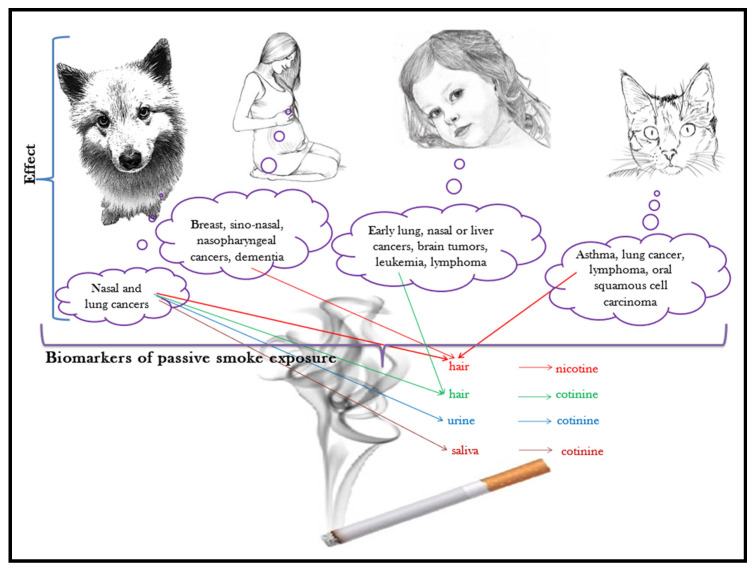
Pets, sentinels of passive smoking.

Maybe now more than ever, having a companion animal nowadays means taking them to accompany us for most of our time: in our houses, in our recreation places, and many times, even at our workplaces. With all the good intentions to provide them shelter and attention, sometimes we expose them to harmful effects of the microenvironment we stay in, and to pollutants that they would not have to face if they were not close by our side. One such pollutant is tobacco smoke. The correlation between passive smoking (second-hand smoking, the inhalation of tobacco smoke by a non-smoker in near proximity of a smoker) and the incidence of respiratory tract diseases and cancers is supported by many studies [80]. The effects of passive smoking are well documented for all those who share living quarters with a smoker, companion animals included [81], as shown in Figure 3.

Through environmental tobacco smoke, companion animals are exposed to nicotine (and its post-inhalation formed metabolite, cotinine), heavy metals, and at least 40 compounds with genotoxic and carcinogenic potential that cause deoxyribonucleic acid (DNA) alteration in the oropharyngeal tissue [43]. Due to these effects, companion animals are considered sentinels for the early destruction of DNA [82].

Cats that live in a smoke-polluted environment for more than 5 years are prone to asthma, lung cancer, lymphoma [83], or oral squamous cell carcinoma [84].

Investigations carried out between 1993 and 2000 in cats demonstrated that the risk rate of malignant lymphoma increases with their exposure to passive smoking, which constitutes alarm signals for the increased risk of non-Hodgkin’s lymphoma among smokers [85]. A year later, Denson [86] observes that the risk of malignant lymphoma is higher in lower socio-economic groups, smokers, who cannot vaccinate cats against infectious feline leukemia, and notes a close correlation between the two diseases [87].

On the other hand, starting from the hypothesis that second-hand smoke leads to an increase in nasal cancer prevalence in dogs, and taking into account factors such as the number of smokers around, the exposure period expressed in years, and the share of time spent inside the house, it was found that dolichocephalic dogs are prone to nasal cancer [88]. This aspect was also emphasized by Bertone-Johnson et al. [89]. Unlike dolichocephalic dogs, mesocephalic and brachycephalic breeds are at risk of lung cancer because they filter less the tobacco smoke [90]. Consequently, cancer of the nose or lung is related to the length of the nose, an extremely important phenotypic feature [91].

The Beagle breed has been for many years a veritable model for human respiratory tract cancer. Histological changes in their lungs are similar to those observed in humans following exposure to cigarette smoke. The inhalation of environmental tobacco smoke causes changes in the macrophages and lymphocytes, anthracosis in the cytoplasm of macrophages with alteration of the respiratory tract cytology [92]. In adult humans, passive smoking is associated with the risk of developing the following different types of cancer: breast, nasal sinuses, and nasopharynx [93]. Studying the impact of passive smoking on the health of pets has extremely relevant connotations for the health of children. Children’s exposure to second-hand smoke is associated with early cancers in the lung [94,95] or nose [96]. Furthermore, the risk of leukemia, lymphoma, liver cancer, and brain tumors increases in children exposed to tobacco smoke [97].

Human medical research to find non-invasive methods to detect exposure to tobacco smoke started about 40 years ago, with the first reporting of nicotine found in human hair, and since then hair became a reliable sampling material for epidemiological studies [98]. In order to track and quantify tobacco through inhalation, absorption, or ingestion, not only nicotine, but also cotinine can be traced in various sample mediums. Cotidine is a metabolic product of nicotine formed inside the exposed organism, it is used as a biomarker, and it can be identified in pets’ blood, saliva, urine, and hair [8]. Several studies have been performed to quantify the presence of both substances in hair, to establish the relationship between the quantity of the inspired smoke and their presence, and to explore the relevance of the findings for human and animal health. As expected, the accumulation of nicotine in human and animal hair depends on the degree of exposure. In the hair of cats and dogs, this conclusion was drawn by comparing the nicotine concentrations found to the owner-reported tobacco smoke exposure [99], considering elements of the hair follicle anatomy and physiology [100], and even comparing it to the quantities found in human hair [101]. About cotinine, Knottenbelt et al. [102] showed that its amounts are similar in canine and children’s hair, representing an early warning sign of future health problems and thus relevant as a valuable biomarker of passive smoking. Yet, Benowitz [103] found that the amount of cotinine varies not only with the exposure time but also with the sex, weight, and age of the pets, with females having higher concentrations of cotinine in their hair than males.

Another non-invasive alternative to assess cotinine is to sample and test canine urine, as demonstrated by Roza and Viegas [92]. This analysis takes into consideration the breed of the dog as well because of the differences in the breathing rate (and thus inhalation quantity) of the heavy breeds compared to the small breeds [104].

The presence of cotinine is quantifiable in the saliva too, particularly in brachycephalic breeds, as an indicator of possible disease among homeowners. Saliva comes into direct contact with the environment in which pets live and is not filtered through the renal system [105].

### 4.2. Pets as Sentinels for Forever Chemicals (Persistent Organic Pollutants and Brominated Flame Retardants)

#### 4.2.1. What Are the Forever Chemicals?

“Forever chemicals” are a group of persistent per- and poly-fluoroalkyl substances (PFAS), which are very resistant to decomposition due to their high electronegativity and dissociation energies of carbon–fluorine bonds [106]. These substances are difficult to detect, especially the PFAS, the perfluorooctane sulfonate (PFOS), and the perfluorooctanoate (PFOA). All these have a considerable bioaccumulation capacity in free-living organisms and humans. They include over 4000 compounds used in plenty of products, including non-stick cookware, stain-resistant fabrics, fast food packaging, and other chemical materials. Once partially broken down, these persist in an omnipresent way in dust, water, and food, they pass through the placenta from mother to fetus, and then through milk ingestion [107].

Since the 1970s, polybrominated diphenyl ethers (PBDEs) have been used in numerous household products, such as electronic goods, synthetic textiles, polyurethane foam, thermoplastics, or commercial materials in the form of tetrabrominated BDE-47, pentabrominated BDE-99 and -100, and hexabrominated BDE-153, 154, octabrominated, brominated congener 209, bisphenol A (BPA) [108]. Their chemical structure is similar to that of the thyroid hormones 3,3′,5-triiodothyronine (T3), and 3,3′,5,5′-tetraiodothyronine (T4), altering their homeostasis [109].

Using chemical additives to prevent combustion and to limit the spread of fire after ignition (flame retardants) represents another health risk for our pets [110]. Polybrominated diphenyl ethers (PBDEs) are brominated flame retardants (BFRs) found in plastics, textiles, electronic castings, and circuitry [111].

Polychlorinated biphenyls (PCBs) include 209 compounds in their group. Their persistence in the environment is markedly high (between 94 days and 2700 years, depending on the molecules) and their accumulation capacity in living organisms, including humans, is high. The category of PCBs is divided into dioxins, furans, and pesticides. Being lipophilic, PCBs tend to accumulate in adipose tissue [112].

Many of the compounds are considered endocrine-disrupting chemicals (EDCs). Through similar sources and routes of exposure, environmental endocrine-disrupting chemicals (EDCs) affect the health of both humans and pets (dogs, cats) and cause multiple perturbances in the body’s homeostasis. The most frequent pathologies triggered by such dysfunctions, including hyperthyroidism, metabolic disorders (like obesity), reproductive problems, renal failure, and even several types of cancer [8].

#### 4.2.2. Comparative Human–Pet Epidemiological Studies on the Effects of Forever Chemicals

Parallel epidemiological studies in humans and animals indicate that exposure to substances in the forever chemicals group is associated with immunotoxicity, cardiac and metabolic diseases, developmental and reproductive effects, cancer, and, more recently, type 2 diabetes [113,114]. Table 4 presents the effects of these compounds on humans and pets.

#### 4.2.3. Effects of Forever Chemicals on Pets

Food ingestion and dust inhalation are the primary sources of human exposure to the persistent chemical compounds [153]. By contrast, inhaling and ingesting indoor dust is the main route of contamination and exposure for pets and especially cats [154]. In cats, dermal exposure leads to oral exposure, as they are active groomers. Licking off polluted dust from their fur is the route favoring the onset of feline hyperthyroidism [116]. The sentinel role for pollution-triggered hyperthyroidism due to indoor dust exposure was established by You et al. [117] and by Brake et al. [118] following their parallel human-feline studies. They found that the quantities of PFAS detected in the serum of cats are higher than in humans, which may be caused by a different metabolic reaction in the two species to those pollutants [119]. Another possible explanation is behavioral. Because the lifestyle of many domestic cats lacks appropriate exercise, their lack of activity may lead to boredom accompanied by excessive grooming. This way the ingestion of PFAS increases, then these act as obesogenic substances, contributing to the high obesity rates in household cats according to Bost et al. [84].

Among canine companions, the effects of chronic exposure to PFAS leads to clinical manifestations, such as constant hair loss, back injuries, numerous fatty tumors, pancreatic disorders, and various gastrointestinal disorders, as was observed by Barnes [138]. Several types of PFAS have been linked to higher or lower than normal cholesterol levels in Beagles and police dogs [117].

Part of the changes disrupting the homeostasis of the pets’ organisms are similar to those occurring in humans, a finding highlighted by the study conducted by Salihovic et al. [141] in 1002 human patients. The effect of PFAS exposure is positively associated with the abnormal activity of several liver enzymes and liver function biomarkers, and it is inversely associated with changes in circulating bilirubin.

According to Norrgran Engdahl et al. [155], 20% of PBDE comes from canned fish when it is part of the diet of people or cats, and the most substantial part of the accumulation of the BDE-209 congener is caused by its presence in the indoor dust. Even though the respiratory exposure of humans and cats to PBDEs compounds is similar, the higher plasmatic levels in cats (caused by dust ingestion while self-grooming) were observed by several authors [156,157]. Researching the impact of the additional exposure, Dirtu et al. [157] sampled and analyzed, in parallel, the plasma of three- to five-year-old children and their feline companions. The results of the BDE 47 to BDE 99 report showed significantly higher values in cats than in children, up to similar concentrations to those found in indoor dust itself. In other words, these cat sentinels show that the ingestion of chemical contaminated dust is a real danger, especially for toothless children who, by coming in to contact with dusty surfaces more frequently, are at a higher risk of ingesting dust than adults. Assessing the BDE 47/BDE 99 congeners’ ratio in the serum simultaneously with the evaluation of this report in the indoor dust provides a valuable method for identifying the cause of contamination. The ratio of BDE 47/BDE 99 < 1 with the predominance of BDE 99 indicates exposure to indoor dust, as it was proven for cats [158].

PBDEs present in indoor dust participate in the occurrence of feline hyperthyroidism. This finding was highlighted by Mensching et al. [159] by the superimposition of feline hyperthyroidism incidence over the exposure of cats to indoor dust through ingestion. While evaluating the relationship between dust ingestion and feline hypothyroidism, Braouezec et al. [160] revealed other aspects as well, such as 20–100 times higher serum values of ΣPBDE in cats compared to adult humans. One of the explanations for this result is the reduced metabolic activity of the cats’ organism in phase I of processing these compounds [155].

The PBDE compounds BDE 209, BDE 207, and BDE 47, and their derivatives can accumulate in organs and tissues, such as the liver, bile, brain, and blood, depending on companion animal species. Comparing canines and felines, a higher accumulation of BDE 209, BDE 207, and derivatives both in tissues and in the brain were found in cats, in which BDE 209, BDE 207, 6OH-BDE 47, 2′MeO-BDE68, and 2,4,6-tri-BPh can cross the hematoencephalic barrier [161]. In the cat liver, the congener BDE 47 is metabolized much faster than BDE 99, which breaks down into OH-HBCD, which is why Zheng et al. [162] consider *Felis catus* to be a promising sentinel for human exposure to HBCD from environmental pollutants.

To observe changes in the homeostasis of the feline organism, Khidkhan [151] conducted a study in parallel by administering a single dose of a mixture of 12 PCBs and small but constant doses of BDE 209 over a year. According to their results, PCBs decrease the serum albumin and total proteins, and produce a decline in testicular weight. As for BDE 209, it was correlated with decreased serum albumin concentrations, lower brain weight, and increased serum levels of high-density lipoprotein (HDL) and triglycerides, hence the conclusion of the authors that chronic exposure may have adverse effects by limiting lipolysis in the liver and initiating lipogenesis from the subcutaneous adipose tissue.

In an attempt to demonstrate canine hypothyroxinemia caused by environmental pollution with PBDEs and PCBs, Lau et al. [128], found that there were no major differences in the concentration of PBDEs and PCBs in dogs diagnosed with hypothyroxinemia compared to healthy ones, only one congener, BDE 183 showing some relevance. In turn, based on the symptoms presented by people exposed to polychlorinated biphenyl (PCB) and the analysis of compounds in the plasma of canine companions Schilling [129] demonstrated that dogs can be considered sentinels that have a greater capacity to accumulate these compounds. Moreover, the symptoms of hypothyroidism in dogs are clearly comparable to the signs of the disease in human patients [127].

Among multiple negative effects on the body, exposure to PCBs increases the risk of various types of cancer, including liver cancer, meningioma, and pancreatic cancer [147]. In support of this statement, Ferrante et al. [163] found that PCBs values resulting from the burning of plastic waste in the open air were higher in dogs diagnosed with cancer than in healthy ones.

Pets, both dogs and cats, can provide information about the decline of human reproductive function during their lifetime. Through chemical sterilization, these companions can provide information on reproductive abnormalities by analyzing the quality of the genital tract tissue and functional abnormalities reflected in quality, quantity, and viability of sperm and ovum through the routine monitoring of fertility simultaneously with the monitoring of contaminants [164].

In 2019, Sumner et al. [165] performed parallel tests on the quality and mobility of human and canine semen that showed that canine companions can represent environmental pollution sentinels of exposure to diethylhexyl phthalate (DEHP) and polychlorinated biphenyl 153 (PCB 153). Two years later, the same authors analyzed the chemical profiles and testicular pathologies in dogs from three regions of Great Britain, one location in Denmark (Copenhagen), and one in Finland (Vantaa). Through parallel investigations on human testicular cancer, the authors demonstrated that environmental influences, and especially the presence of diethylhexyl phthalate (DEHP), polybrominated diphenyl ethers (PBDE), and polychlorinated biphenyls (PCB), have negative repercussions on male reproductive function, such as the poorer semen quality and an increased incidence of testicular cancer [145].

The conclusion published by the Environmental Working Group [79] states that blood values showing 2.4 times higher accumulation of perfluorochemicals from stain- and grease-proof coatings in canine companions, and 23 times higher PBDEs in cats are valuable warnings about household pollution dangers.

Chemicals found in lawn care products with carcinogenic potential were identified by Knapp et al. [166] by administering herbicides [2,4-dichlorophenoxyacetic acid (2,4-D), 4-chloro-2-methyl phenoxy propionic acid (MCPP), dicamba] and then analyzing the urine of companion animals. The authors conclude that our pets can serve as sentinels for potentially harmful environmental exposures to humans. Not only can urine be considered a biomarker for the determination of pesticides, but also the feces, to detect 2,4-dichlorophenoxyacetic acid (2,4-D) [167] and other at least 13 different PFAS compounds [106].

Acrolein, used as a pesticide but also used to obtain acrylic acid necessary for the manufacture of plastic, paints, adhesives, and superabsorbent polymers for diapers [168], can represent a potential health risk, especially since the values found in dogs and cats exceed the permitted thresholds [169]. Urinary biomarkers for acrolein and arsenic have a 2.8–6.2-fold increase in canine companions compared to owners exposed to the same mutagenic household chemicals. The preliminary tests performed by Craun et al. [170] are part of a larger project that attempts to quantify the mutagenic potential of household substances in dogs with urothelial carcinoma (UCC), which could play a sentinel role in identifying the owners’ exposure to carcinogens and mutagens correlated with the occurrence of bladder cancer.

#### 4.2.4. New Non-Invasive Ways to Measure Exposure to Forever Chemicals in Pets

A simple method to detect exposure to environmental contaminants, especially flame retardants, tobacco products, and PAHs, is the wearing of silicone wristbands, tags, and collars [122]. Silicone wristbands represent a promising passive tool to support epidemiologic studies that characterize exposure to organic contaminants, especially because the results can be obtained much faster compared to years of latency period in many chronic diseases triggered by these pollutant agents [121]. The use of these devices reduces the costs and time needed for sampling compared to other methods, such as dust collection and serum investigation [171]. For this reason, some testing methods have gradually been replaced by the examination of silicone collars and wristbands that can quantify the level of exposure to organic contaminants and especially to herbicides. These devices can detect with certainty 72 existing compounds, some of them (such as fipronil sulfides, the degradation products of fipronil, and DDE’, the metabolites of DDT metabolites) being considered endocrine disruptors. In addition, they are cheap devices, easy to wear, non-invasive, and silicone has the same potential to absorb chemicals as human or animal cell membranes [172]. More than that, between the levels of contaminants from the analysis of the tags worn by the canine companions and urinary metabolites (validated urinary biomarkers), significant correlations were obtained for several organophosphorus esters, including permethrin and N,N-diethyl-meta-toluamide (DEET) [121].

In fighting fires, firefighters are exposed to numerous compounds that raise the rate of cancer among them. The wearing of silicone tags by their service dogs revealed their exposure to 18 PAH, di-n-butyl, diisobutyl phthalate, guaiacol, and DEET classified as having possible carcinogenic effects [173]. Poutasse et al. [174] used the silicone devices worn by firefighter service dogs for temporized determinations found that some endocrine-disrupting chemicals (EDC) mainly come from household products and environments, and less from work situations of firefighting.

The negative animal health impact of exposure to numerous flame retardants present inside homes, predominantly BDE 47 and BDE 99, was revealed by the parallel measurement of organophosphate ester (OPE) metabolites in urine and silicone tags, which demonstrated, once again, these tags represent valuable devices in the assessment of household pollution [115]. The study of silicone collars worn by cats, and similar wristbands by owners, have demonstrated that cats can be considered sentinels of human exposure to numerous contaminants, as silicone collars are more sensitive than wristbands to many PAH compounds [175].

The quantitative evaluation of flame retardants (FRs) that may contribute to feline hyperthyroidism was carried out using passive silicone pet tags, useful in assessing the exposure to many compounds, including the detection of Tris (1,3-dichloro-2-isopropyl) phosphates (TDCIPP). Research performed by Poutasse et al. [176] on silicone devices worn by 76 cats found that Tris (1,3-dichloro-2-isopropyl) phosphate (TDCIPP) associated with air freshener use has higher concentrations in hyperthyroid cats, and is also associated with higher concentrations of free thyroxine (fT4), and total T4 (TT4).

## 5. Pets as Sentinels and Models in Comparative Human–Pet Oncological Studies

**Figure 4:**
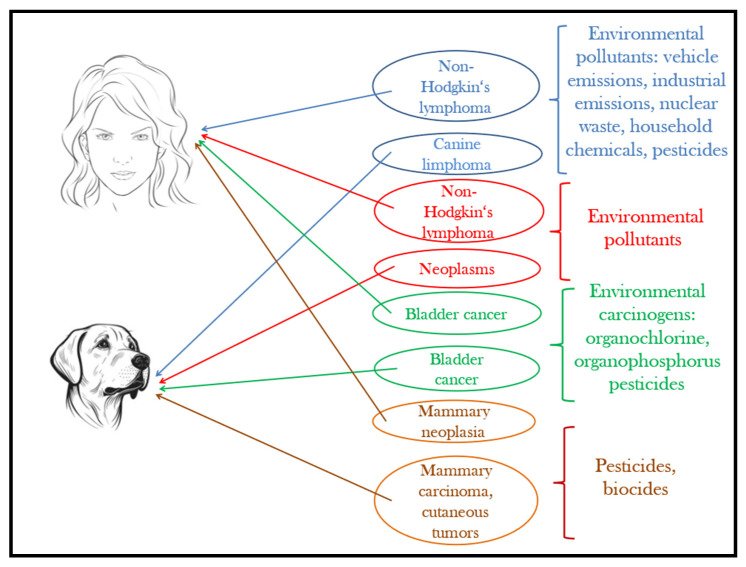
Pets, sentinels of environmental carcinogens.

Although pets have a shorter lifespan than humans, they develop similar disorders and homeostatic changes to humans under the effect of the same environmental factors and pollutants [177]. Figure 4 provides an illustration in this regard. The advances of today’s medical technology give the unprecedented possibility to perform complex epidemiological studies on our companion animals to understand the effects of increasing pollution and extrapolate the results on the human species. As reported by Ruple et al. [178], the overall incidence of cancer is 10 times higher in dogs, and the latency period is much shorter compared to humans, where initial signs of disease can appear after 30–35 years. The analysis of spontaneous tumors in dogs is not only a broad area for exploring pathogenesis, but also it significantly reduces the need for laboratory animal testing [179,180].

Despite the availability of more than 1000 strains of transgenic mice currently used in medical research [181] for environmental and genetic factors that trigger the onset of human cancer, studying the disease in dogs is more appropriate. One of the reasons is that we have over 650 million base pairs of ancestral DNA sequences in common with the domestic dogs, and their orthologous genes share a 75% similarity with that of ours [182].

Based on epidemiological investigations carried out in the city of São Paulo, Kimura et al. [183] found a strong association between the spatial distribution of canine lymphoma and non-Hodgkin lymphoma. The results suggest that in the pathogenesis of lymphomas, environmental pollutants caused by vehicle emissions and heavy traffic can play an important role.

Another investigation showing that similar human–pet environmental conditions are positively correlated with neoplasm occurrence in dogs and non-Hodgkin’s lymphoma in humans was carried out by Pinello et al. [184] over five years in the Greater Porto area (north-western Portugal). The authors concluded that male canine companions can especially be used as sentinels to environmental pollutants associated with this type of cancer. In humans, men are more likely to have this type of lymphoma [185].

Canine lymphoma has also been positively correlated with the habitat that pets share with their owners, with a higher occurrence near industrial areas, or when owners repeatedly use certain types of paints at home [186]. Craun et al. [187] later added other risk factors. Investigating 63 boxer dogs, they highlighted the increased incidence of lymphomas near nuclear power plants, chemical product suppliers, and crematoriums. Baioni et al. [188], comparing the cases of malignant tumors in dogs from the north-west of Italy based on the Canine Cancer Registry of Piedmont and those existing among the human population, reached the conclusion that the records represent a reliable reference for comparative studies in the risk detection of carcinogenic environmental contaminants to which the human community is exposed.

Another documented research draws attention on the morbidity rates due to canine bladder cancer, which, according to Hayes et al. [189], may represent a sentinel situation and help in the early detection of environmental carcinogenic hazards for the people living in polluted industrial areas.

Human health problems associated with persistent organic pollutants are related to immune suppression, genotoxic effects, or cancer [80]. Cumberbatch et al. [190] specifies that bladder cancer ranks 9th in occurrence and 13th in annual deaths for humans worldwide.

Several authors consider that transitional cell carcinoma can be associated with organochlorine, organophosphorus pesticides, tobacco, and other household products [191]. The potential risk of transitional cell carcinoma of the bladder, particularly in obese dogs, is related to the use of herbicides and insecticides [192]. Similarly, Glickman et al. [193] noted that treating the lawn with phenoxy herbicides leads to a considerable increase in transitional cell carcinoma (TCC) in the urinary bladder in Scottish Terriers. Bladder cancer was diagnosed following histological evidence of transitional cell carcinoma in 89 dogs exposed to insecticides used to control ticks and fleas. These acaricides and insecticides, which accumulate in the fat deposits of the body, have a high potential to trigger tumoral processes. As Mutsaers et al. [194] describes, there are the following similarities between human and canine bladder cancer symptoms: hematuria, dysuria, urinary tract infection, immunoreactivity to TAG-72 antibodies, and most frequent metastatic sites (regional lymph nodes and lungs). The occurrence of metastases is slightly different between the two species; for 20% of dogs, 5–10% of humans are diagnosed with them.

Another unfortunate consequence of human exposure to pesticides is the high risk of mammary neoplasia [195]. The exposure of pets to the same environment claims them as sentinels because of high mammary carcinoma incidences. The observations made by Andrade et al. [196] concluded that pyrethroid pesticides directly affect hormonal homeostasis, especially that of estrogen, and indirectly lead to cell proliferation or apoptosis, increasing or reducing the number of mammary epithelial cells. In 33.3% of canine females with mammary carcinoma, insecticides were found in adipose tissues.

By evaluating the serum, mammary tissue, and adjacent mammary adipose tissue from female dog patients diagnosed with mammary cancer, Gautam et al. [197] demonstrated the presence of 14 different pesticides in much higher concentrations than in samples from healthy dogs. Among them, γ-HCH was most commonly highlighted, while Heptachlor, aldrin, and p, p1-DDT had a lower frequency. A positive correlation was found between the presence of tumors and the age of females. The share with the highest diagnostic rate was assessed in the four- to eight-year-old group, followed by the category between 8 and 12 years of age.

In contrast to other results in which levels of organochlorine pesticides in plasma (OCP) were higher in cats, the results of the study conducted by Yavuz et al. [198] in 15 cats and 21 dogs revealed much higher levels of OCP (α-hexachlorocyclohexane (HCH), β-HCH, γ-HCH, hexachlorobenzene (HCB), aldrin, 2,4′-dichlorodiphenyltrichloroethane (2,4′-DDT), 4,4′-DDT, 2,4′-dichlorodiphenyldichloroethylene (2,4′-DDE), and 4,4′-DDE among canine companions.

Our companion animals do their best to warn us about the potential dangers of exposure to 2,4-D, which increases the risk of malignant lymphoma in dogs, is associated with non-Hodgkin’s lymphoma in humans, and is also a danger to children’s health [199].

In the cat, considered much less sociable than the dog but a true bioenergetic device [200], the frequency of oral squamous cell carcinoma (SCC) occurrence is relatively high. The risk of this disease may be heightened by wearing flea collars, up to five times more than in congeners without a collar [201]. There are multiple causes of oral squamous cell carcinoma occurrence in humans, including smoking, but studies undertaken by Devianto et al. [202] have demonstrated that there is a more pronounced affinity among farmers exposed to sunlight and pesticides for the disease to be triggered.

Even though the results were not extremely conclusive, Enriquez et al. [203], observed that cutaneous tumors in dogs are proportional to the rate of pesticides or biocides use by the owners.

Fipronil from the products used as external antiparasitic against fleas and ticks has a residual time of about two months after administration and it can irritate the skin of pets. Consequently, cats and dogs may exhibit lethargy, incoordination, dilated pupils, facial swelling, and convulsions [204]. Human exposure to these substances can lead to neurotoxicity, liver and kidney damage, skin irritation, eye damage, reproductive effects, and cancer [205]. 

Although pets are already considered our “canaries” for a number of carcinogens, Rabinowitz et al. [76] note that canine companions have a yet unrevealed potential to detect the multitude of compounds and assist in finding means of preventing human cancer.

## 6. Pets as Sentinels in the “One Health” Approach

When considering environmental pollution, we have to be aware that the concept is broader than just inorganic or organic compound contamination. Microorganisms (bacteria and viruses) can be considered dangerous pollutants, too. In this regard, our canine companions represent biomonitors of pathogen exposure [206] since they are susceptible to various infections. People, animals, the environment, and the interdependence between these three components underpin the “One Health” approach proposed by the European Commission [207]. At present, more than ever, zoonotic diseases have a rapid spread, and pets, especially canine companions, can be considered sentinels for disease prediction [208,209].

In some cases, the dogs have multiple roles in this regard, as described in a recent paper by Balboni et al. [210] about leptospirosis caused by *Leptospira* spp., a zoonotic disease with possible fatal effects in many species. Besides being susceptible to the disease, dogs can also be potential hosts and an important sentinel species for the dangerous microorganism.

Another pathogen classified as a possible biological weapon is *Coxiella burnetti*, the causative agent of Q fever, a zoonotic disease with serious debilitating potential. According to Orr et al. [211] this disease spreads alarmingly among dogs in eastern and central Australia recently. The danger is greater among pig-hunting dogs; almost one-fifth of the tested dogs were found to be seropositive, and out of them the sterilized dogs were more susceptible than non-sterilized ones. The authors conclude that dogs can be considered sentinels of *C. burnetti* exposure, providing an early warning for humans about the pathogen’s existence in a certain area.

Dogs can be considered sentinels for plague caused by *Yersinia pestis*. Due to the rapid serological response to the plague bacillus among canine companions, they can be deemed useful sentinels for the detection of plague in areas frequented by humans [212]. The research led by Rajerison et al. [213] demonstrated that, parallel analyzes in humans and several animal species show that dogs have good sensitivity and performance in evaluating the SIgT test, for the detection of total Ig (IgT) anti-F1, being considered sentinels.

Dogs can also provide useful information on the risk of human exposure to *Bartonella* spp., which is why Henn et al. [214] mention that they can serve as a sentinel system for surveillance in endemic areas.

For human and horse meningoencephalitis produced by the West Nile virus becoming more and more endemic around the globe, dogs can represent reliable alternative sentinels by affordable serologic analyses [215]. Komar et al. [216] note that young animals are more susceptible, and Kile et al. [217] state that seropositivity is higher among family dogs kept outdoors than for those kept indoors (exposure to the disease vector). Stray cats can also be used as sentinels in West Nile virus surveillance [218].

The risk of *Trichinella spiralis* infection, especially in people consuming game meat, can be monitored by regularly serum testing the game hunting dogs to map the parasite’s spreading areas. The same method could also be implemented to identify antibodies that cause risk-related diseases of human health, such as the following: ehrlichiosis, leishmaniasis [219], tularemia, and Rocky Mountain spotted fever [220].

### Pets as Sentinels outside the Scientific Definition

In the introduction of this review, we stated, “Sentinels are organisms whose characteristics change as a result of acute or chronic effects that can be evaluated through serial surveillance […] to identify potential health hazards to humans or other animals” [4]. Outside the scientific definition and said simply, sentinels watch over dangers and warn others. Although it does not produce quantifiable changes in their organism and the warning is behavioral, companion animals are sentinels of human health outside the definition too, through their ability to perceive and signal certain health problems in humans. The developed sense of some dogs to detect early forms of cancer is already known and enhanced by specific training in many parts of the world. Cancer cells in affected humans can produce and release odor signatures, volatile organic compounds that dogs can smell out from skin, breath, urine, and sweat, and can alert humans about their presence [221,222]. They are considered medical detection dogs because they can warn about early-stage melanoma or colorectal, lung, ovarian, prostate, and breast cancers [223,224].

In many circumstances, canines are considered helpers, protectors, and service providers. Seizure-alert dogs are trained to warn even before a person has a seizure, one of the most common neurological disorders in the world [225,226]. They can naturally detect the change in the smell emitted by the human before the onset of a seizure or even intervene to avoid it [227,228].

A less-known benefit of cohabitation with companion animals is that they support people’s health by exposing them to animal-specific microorganisms. For example, the modern treatment for human asthma, atopic dermatitis, rhinitis, certain cardiovascular diseases, obesity, and even depression, proposes the microbiome-based approach, based on exposure to mixed microbes through the symbiosis created between humans and their pets [229].

## 7. Conclusions

Our canine and feline companions can act as sentinels for a tremendous number of pollutants and contaminants, which have similar negative effects on their soundness as on the health of humans, especially children.

Investigating in parallel the occurrence of certain diseases in humans and pets, which have a high incidence in given geographic areas, can expand the field of medical research and can aid in finding more affordable solutions both in terms of sampling time and data processing.

The collaboration between human and veterinary medical laboratories represents an advantage in preventing diseases caused by environmental contaminants. Based on the results of such collaboration, the environmental and other public authorities can be warned, solutions can be found, or activities can be implemented in order to improve the quality of the environment in which we all live.

Despite their widespread use, the possible health hazards posed by several substances and products are not known by the general public. Many times, unknowingly, we pollute the macro and microenvironment, exposing ourselves, our children, and our pets to harmful consequences. Gaining more knowledge on the composition and effects of potential household pollutants can lead to more responsible environment-protective behavior by both pet owners and those not owning companion animals.

Although most of the time, microbiological contaminants (microorganisms) are not regarded as pollutants of the environment, the sentinel role of companion animals is valuable within epidemiological studies of both existing and emerging diseases. This is especially true in the current context of intense international transportation (possibly translocating pathogens) and climatic changes (allowing pathogen and vector survival in naïve areas).

We, the authors, consider that general awareness has to be raised in all these aspects, and we hope to contribute with a grain of knowledge to this task through our review paper.

## 8. Future Directions

In the light of the scientific research reviewed in this paper, the benefits of centralized actions became clear. Parallel analyses in humans and companion animals to monitor national and international level mortality and morbidity linked to the degree of environmental pollution, authority level interdisciplinary collaborations in finding strategies to reduce the negative impact of polluted environments and to prevent further contamination are highly desirable. Several similarities shared by humans and pets exposed to the same pollutants are scientifically proven to date and highlighted in the present review. Besides their theoretical value as research-based knowledge, these can support efficient and low-cost environmental and health monitoring policies, especially in developing countries. With the involvement of legal entities, pets can be used as sentinels through public policy in an official way. With or without these very much-needed formal actions, we all can and should contribute, in our own quality: as a scientist team, as a single researcher, or as an informed and responsible citizen, regardless. In our consumer society, the conscious choice to use or not the available products and to care for the ways we use these can and do matter—for our pets, for our children, for ourselves, and, eventually, for our planet Earth.

## Figures and Tables

**Table 1:** Human and small animal pathologies promoted by metalloid or heavy metal toxic exposure.

Heavy Metal/Metalloid	Pets	Effects in Pets	References	Effects in Humans	References
Asbestos	Dogs	Canine malignant mesothelioma Pleural mesothelioma (more frequently than pericardial and peritoneal)—poor prognostic Pleural effusions, especially in old dogs, with males more prone than females	[13] [14] [15]	Human mesothelioma Pleural mesothelioma (more frequently pleural effusions) Peak incidence occurs in the 5th and 6th decades of life	[16] [17] [18]
Chromium (Cr)	Dogs	Cardiac impairment, oxidative damage, altered ATP-aze content	[19]	Airway irritation, obstruction, and cancers Allergic dermatitis Dose dependent renal damage Gastrointestinal and hepatic impairment Hypoxic myocardiac changes Intravascular hemolysis	[20]
Arsenic (As)	Dogs Cats	Ulcerative dermatitis Myocarditis Bladder cancer Chronic renal failure	[21] [22]	Hyperpigmentation and keratosis Ischemic heart diseases Renal diseases Bladder cancer, skin, lungs, liver, kidney cancer Kidney damage	[23] [24] [25]
Cadmium (Cd)	Dogs Cats	Disrupted male reproduction Impaired pancreatic function Decreased bone-formation rate Chronic renal failure	[26] [27]	Altered male reproduction (hormonally and functionally) Disturbed calcium metabolism: osteopetrosis, osteomalaecia Itai-Itai disease (renal tubular dysfunction and osteomalaecia) Kidney failure	[28] [29] [30]
Lead (Pb)	Dogs Cats	Functional forebrain dysfunction and cortical blindness Anemia Epileptic seizures Bone sclerosis Chronic myocarditis chronic Renal failure	[31]	Central and peripheral nervous system impairment Hematopoiesis problems, microcytic anemia Gastrointestinal disturbances Renal failure.	[32] [33] [34]
Mercury (Hg)	Cats	Similar neurological symptoms to Minamata disease: ataxia, weakness, loss of balance, and motor incoordination	[35] [36]	Neurological symptoms: uncontrollable tremors, muscular incoordination, slurred speech, partial blindness	[37]

**Table 2:** Average values of heavy metals found in the blood and certain organs of clinically healthy ^†^ pets from different polluted geographical areas.

Country	U.M.	Heavy Metals (Mean ± SD)					Analyzed From	No. of Samples	Species	References
		Pb	Cd	Cr	Hg	As				
Korea	μg/mL	0.68 ± 0.19	0.21 ± 0.01	0.66 ± 0.15	1.10 ± 0.49	-	Serum	204	Dogs	[43]
Zambia	μg/L	271.6 ± 226.9	1.5 ± 1.6	67.2 ± 75.4	-	5.2 ± 4.5	Blood	120	Dogs	[44]
Italy (Campania)	mg/kg	0.321 ± 0.198	0.093 ± 0.079	-	0.054 ± 0.044	-	Liver	38	Dogs	[45]
		0.293 ± 0.231	0.259 ± 0.238	-	0.040 ± 0.021	-	Kidney			
Italy (Naples)	mg/Kg	0.256 ± 0.130	0.098 ± 0.063	-	-	-	Liver	290	Dogs	[46]
		0.147 ± 0.081	0.302 ± 0.212	-	-	-	Kidney			
		0.268 ± 0.107	0.101 ± 0.054	-	-	-	Liver	88	Cats	
		0.189 ± 0.102	0.355 ± 0.144	-	-	-	Kidney			
Italy (South Sardinia)	ng/mL	81.4 ± 16.6	52.2 ± 14.0	-	-	139 ± 39	Ovaries	26	Cats	[47]
		20.4 ± 3.6	19.7 ± 4.0	-	-	21.7 ± 4.2	Ovaries	21	Dogs	
Italy (North Sardinia)	ng/mL	51.1 ± 17.9	26.4 ± 5.5	-	-	107 ± 61	Ovaries	14	Cats	
		12.2 ± 5.2	12.2 ± 1.8	-	-	21.8 ± 3.9	Ovaries	24	Dogs	
Poland		2.829 ± 3.490 ** 1.55 ± 1.71 **	0.105 ± 0.067 ** 0.096 ± 0.074 **		0.0020 ± 0.0013 * 0.0027 ± 0.0022 **		Cartilage and compact bone Spongy bone	24	Dogs	[48]
Poland	μg/mL	0.419 ± 0.027	0.302 ± 0.049	0.244 ± 0.016	-	0.637 ± 0.277	Serum	48	Dogs	[49]
Spain	μg/mL	0.55 ± 0.60	-	2.73 ± 2.54	0.24 ± 0.22	1.86 ± 1.44	-	42	Dogs	[50]
Jamaica West Indies	μg/mL	2.83 ± 0.40	-	-	-	-	Serum	63	Dogs	[51]
New Zealand	μg/mL	0.23 ± 0.66	-	-	-	-	-	271	Dogs	[52]
		0.21 ± 0.05	-	-	-	-	-	113	Cats	

^†^ One of the referenced papers [46] reports the heavy metal concentrations found in the tissues of animals deceased in an incident. U.M.: unit of measurement; * lower values than in humans; ** higher values than in humans.

**Table 3:** Mean values of heavy metals obtained by testing pet hair samples.

Country	UM	Heavy Metals (Mean ± SD)					AM	No. of Samples	Species	References
		Pb	Cd	Cr	Hg	As				
Macedonia	μg/kg									
Veles		930.15 ± 516.03	54.28 ± 12.77	-	-	-	AAS	11	Dog	[59]
Bitola		715.66 ± 293.80	42.65 ± 25.41	-	-	-		22	Dog	
Prilep		525.63 ± 253.91	27.82 ± 8.31	-	-	-		11	Dog	
Macedonia	μg/kg									
Delcevo		579 ± 478.29	68.57 ± 59.95	-	-	-	AAS	18	Dog	[60]
Probistip		1061.38 ± 564.02	26.86 ± 23.30	-	-	-		20	Dog	
Veles		1099.02 ± 593.01	171.54 ± 179.53	-	-	-		17	Dog	
Prilep		370.57 ± 288.39	21.65 ± 10.64	-	-	-		18	Dog	
Bitola		687.05 ± 482.82	66.04 ± 73.78	-	-	-		21	Dog	
Australia	mg/kg^−1^ DW	1.19 ± 3.11	-	0.85 ± 1.42	0.13 ± 0.11	0.08 ± 0.06	AAS	36	Dog	[61]
Argentina	mg/g DW^−1^	-	-	-	-	24 ± 2	TXRF technique	-	Dog	[62]
Portugal	μg/g^−1^				24 ± 2.4		TXRF	50	Dog	[63]
Portugal	ng/g^−1^	-	-	-	24.16–826.30		TXRF technique	26	Dog	[64]
Poland	mg/kg^−1^	-	-	-	0.025 ± 0.020		AAS	85	Cat	[65]
Iran	ng/g DW	-	-	-			AAS	40	Wild cats	[66]
North-west		-	-	-	735 ± 456					
North		-	-	-	568 ± 381 692 ± 577					
Center		-	-	-	1303 ± 1306 376 ± 162					
North-east		-	-	-	1517 ± 1888					
West		-	-	-	231 ± 89					
Japan	ppm	-	-	-	7.40 ± 2.93		AAS	12	Male cat	[67]
		-	-	-	7.45 ± 1.28			29	Female cat	
		-	-	-	0.99 ± 0.23			16	Male dog	
		-	-	-	0.66 ± 0.10			18	Female dog	
Alaska	ng/g				1822.4 ± 1747			-	Sled dog	[68]

UM—unit of measurement; DW—dry weight; AAS—atomic absorption spectrometry; AM—analysis method; TXRF—total reflection X-ray fluorescence analysis.

**Table 4:** Pathological effects of exposure to forever chemicals (persistent organic pollutants and brominate fire retardants) reported in humans and companion animals.

Forever Chemical Compound	Effects in Pets	Effects in Humans	References
PFAS: producing endocrine thyroid toxins	Cats: hyperthyroidism, hyperplastic or adenomatous thyroid nodules, abnormal thyroid, and thyroid-stimulating hormone levels	Toxic nodular goiter, abnormal thyroid and thyroid-stimulating hormone levels	[108,115,116,117,118,119,120,121,122,123,124,125,126]
PBDEs	Dogs: hypothyroidism	Thyroid toxicity	[127,128,129,130]
PFAS: acting as obegosenic substance	Cats: obesity	Child and adult obesity	[84,123,131]
PFAS: acting as diabetogenic	Cats: type 2 diabetes	Gestational diabetes mellitus, type 2 diabetes, pancreatic islet amyloid deposition	[131,132,133,134,135,136,137]
PFAS	Dogs: fatty tumors, pancreatic dysfunction, gastrointestinal disorders	Chronic liver disease, fatty liver disease, hepatotoxicity	[113,138,139,140]
PFOA, PFOS	Dogs (Beagles and police dogs): lowered cholesterol	Lowered direct bilirubin, changes inβbilirubin, βalt, βalp, increased alanine aminotransferase, Alcaline phosphatase, aspartate aminotransferase, gamma glutamyl transferase	[105,107,141,142,143,144]
PFNA, PFDA	Dogs: increased circulating cholesterol	High blood cholesterol	[107,142,143]
PBDEs PCBs	Dogs: poor semen quality, testicular cancer	Disruption of reproductive function and infertility, decreased sperm counts, and genital malformations	[42,145,146]
PCBs	Dogs: liver cancer, meningioma, pancreatic cancer, mammary cancer in females	Melanomas, liver and gall bladder cancer, biliary tract, gastrointestinal tract, brain cancer	[120,140,147,148,149,150]
PCBs	Cats: lower brain weight at birth	Decreased birth weight and head size	[117,151,152]

PFAS—per- and poly-fluoroalkyl substances; PBDEs—polybrominated diphenyl ethers; PFOA—perfluorooctanoate; PFOS—perfluorooctane sulfonate; PFNA—perfluorononanoic acid; PFDA—nonadecafluorodecanoic acid; PCBs—polychlorinated biphenyls.
